# Impact of endurance training on mitochondrial H_2_O_2_ production and NRF2 levels in different rat organs

**DOI:** 10.3389/fmolb.2025.1653162

**Published:** 2025-10-16

**Authors:** Lukasz Galganski, Krzysztof Wojcicki, Wieslawa Jarmuszkiewicz, Jerzy A. Zoladz

**Affiliations:** ^1^ Mitochondrial Biochemistry Research Group, Faculty of Biology, Adam Mickiewicz University in Poznan, Poznań, Poland; ^2^ Chair of Exercise Physiology and Muscle Bioenergetics, Faculty of Health Sciences, Jagiellonian University Medical College, Krakow, Poland

**Keywords:** endurance training, mitochondrial ROS formation, NRF2, oxidative stress, multi-organ adaptive response

## Abstract

**Background:**

In recent years, increasing attention has focused on the effect of exercise on redox balance and the expression of nuclear factor erythroid 2-related factor 2 (NRF2), widely recognized as the master regulator of antioxidant defense mechanisms. However, surprisingly little is known about how physical training influences H_2_O_2_ production and NRF2 expression across various vital organs.

**Methods:**

We investigated the effects of endurance training on the oxidative capacity, reactive oxygen species production, and antioxidant defense of various body organs in rats. Sixteen 4-month-old male Wistar rats were randomly assigned to either an endurance training group (8 weeks of treadmill running, *n* = 8) or a sedentary control group (*n* = 8).

**Results:**

In the endurance training group, maximal oxidative activity increased in all examined tissues (lung, brain, liver, and hind limb skeletal muscle) except the heart. Under phosphorylating conditions, H_2_O_2_ production remained unchanged in all tissues except the heart, where it increased. Under non-phosphorylating conditions, H_2_O_2_ production increased only in the liver and heart. In all tissues, H_2_O_2_ production was consistently lower under phosphorylating than non-phosphorylating conditions. The level of malondialdehyde, a marker of oxidative damage, did not increase in the examined tissues, except the lungs, where it even decreased. Superoxide dismutase 1 levels increased in the lung, brain, and skeletal muscle, but decreased in the heart and remained unchanged in the liver. NRF2 protein levels were significantly elevated in all examined tissues, accompanied by an increase in glutathione reductase levels.

**Conclusion:**

Given the cytoprotective capacity of NRF2, we postulate that the NRF2-regulated adaptive multi-organ response may play a key role in the widely described beneficial effects of physical activity on various body organs and body health.

## 1 Introduction

Mitochondria play a key role in energy production in various organs of the human body via the process of oxidative phosphorylation (OXPHOS) (Lima et al., 2022; Vercellino and Sazanov, 2022). Some organs, such as the brain, heart, and skeletal muscle, are highly dependent on energy supplies in the form of ATP produced via OXPHOS at rest and during moderate-intensity exercise (Dunker et al., 2019; Hargreaves and Spriet, 2020). It is well documented that physical training increases mitochondrial protein biogenesis in rat limb skeletal muscle (Holloszy, 1967) as well as in human leg skeletal muscle (Morgan et al., 1971; Wibom et al., 1992; Zoladz et al., 2022), but its effects on mitochondrial biogenesis and function in other tissues, such as the heart, brain, lungs, or liver, are much less known. It has long been known that mitochondria produce two main oxidants (Loschen et al., 1971; Boveris and Chance, 1973): superoxide anion radicals (O_2_^•−^) and hydrogen peroxide (H_2_O_2_) (Brand et al., 2004; Kowaltowski et al., 2009; Jarmuszkiewicz et al., 2015). Davies et al. were the first to demonstrate that intense physical exercise increases the production of reactive oxygen species (ROS) in skeletal muscle and liver (Davies et al., 1982), which has since been confirmed by others (Powers and Jackson, 2008; Powers et al., 2024b). It is widely accepted that excessive ROS production leads to oxidative stress (Zhou et al., 2024), resulting in the dysfunction of many body organs (Nathan and Cunningham-Bussel, 2013; Ma et al., 2017; Chen et al., 2018).

Opposite to inactivity, physical activity improves the functioning of many body organs and significantly reduces the risk of many chronic medical conditions (Pedersen and Saltin, 2015; Warburton and Bredin, 2017; 2019). Increasing evidence suggests that exercise-induced increases in ROS (especially H_2_O_2_) can trigger a range of adaptive responses via redox signaling that beneficially affect the function of various organs (Shadel and Horvath, 2015; Sies and Jones, 2020; Powers et al., 2024b; Sies, 2024; Zhou et al., 2024). Therefore, exercise-induced ROS production is believed to be essential to achieve the full benefits of exercise-induced skeletal muscle adaptation (Powers et al., 2024b). Nonetheless, little is still known about the effect of endurance training on mitochondrial H_2_O_2_ production in different organs, such as the lung, brain, liver, heart, and skeletal muscle, as well as on the antioxidant defense status in these organs after physical training.

It is well-documented that muscles have a potent antioxidant system (Powers et al., 2024a; Zhou et al., 2024), which effectively protects them from oxidative stress in many conditions. Indeed, exercise increases the levels of several antioxidant enzymes, such as superoxide dismutase 1 (SOD1), glutathione peroxidase, glutathione reductase, and catalase (Powers et al., 2024a). In recent years, the primary focus of antioxidant defense research has been nuclear factor erythroid 2-related factor 2 (NRF2), considered the master regulator of antioxidant responses (He et al., 2020; Gao et al., 2021; Esteras and Abramov, 2022; Powers et al., 2024a; Zhou et al., 2024). It should be noted, however, that most studies to date on the effects of physical activity or training on redox control have focused primarily on skeletal muscle (Powers and Jackson, 2008; Powers et al., 2024a; Zhou et al., 2024). In recent years, knowledge about the effects of physical exercise on ROS production in some vital body organs such as the brain, heart and liver has also increased (Davies et al., 1982; Radak et al., 2016; German et al., 2017; Quan et al., 2020; Dominiak et al., 2022), but knowledge about the effects of exercise on redox regulation and the functioning of these organs is still very limited. Surprisingly, little is known about the effects of physical training on mitochondrial H_2_O_2_ production in the lung, brain, liver, heart, and hind limb skeletal muscle in studies using the same animals. Furthermore, no data have been published on the effects of physical training on NRF2 expression in these organs.

Therefore, this study primarily aimed to determine − for the first time, to the best of our knowledge − the effect of forced endurance training (performed on a treadmill) on NRF2 expression in the lung, brain, liver, heart, and hind limb skeletal muscle in rats. It also aimed to determine the impact of physical training on (i) mitochondrial H_2_O_2_ production under phosphorylating and non-phosphorylating conditions, (iii) the level of malondialdehyde (MDA, a marker of lipid peroxidation), SOD1 and glutathione reductase (markers of antioxidant defense), (iv) mitochondrial biogenesis, and (v) the maximal activity of cytochrome *c* oxidase (COX) and citrate synthase (CS, markers of mitochondrial oxidative capacity) in the homogenates of the studied tissues.

## 2 Materials and methods

### 2.1 Rat endurance training

Sixteen 4-month-old male Wistar rats were randomly assigned to an endurance training group (*n* = 8) or a sedentary control group (*n* = 8). During this study, the rats were kept in standard laboratory cages (two per cage) under controlled conditions of temperature (22 °C ± 2 °C), humidity (55% ± 10%), 12/12-h light/dark cycle, and free access to a standard rat food, providing a balanced diet, and tap water. The experimental protocols regarding training, surgical procedures, and animal care were approved by the Local Ethics Committee for Animal Experiments in Poznan, Poland (Permit Number: 15/2013) and consistent with the guidelines of Directive 2010/63/EU of the European Parliament and of the Council of 22 September 2010 on the protection of animals used for scientific purposes. Every effort was made to minimize the suffering of the experimental rats.

The 8-week training program consisted of five training sessions per week on a small rodent treadmill (Exer 3/6 M treadmill; Columbus Instruments, Columbus, OH, United States), as previously described (Zoladz et al., 2016). The running belt was set horizontally with an inclination of 0°. In the first week, the training sessions lasted 20–30 min and were intended to familiarize the rats with running on a treadmill at different speeds (20–30 m/min). Then, the training sessions were extended to 40 min, and the basic running speed was set at 30 m/min until the end of the training program. During the first 2 weeks, the basic running speed was increased transiently to 40 m/min for 20 s every 10 min. From the fifth week, the running training on the treadmill lasted 60 min, and the running speed was increased to 40 m/min every 10 min. The duration of higher running speed was gradually increased from 20 s in the sixth week to 40 s in the eighth week.

The rats were sacrificed by decapitation 22–24 h after the last training session. The hind limb skeletal muscle and the lungs used in this study were from rats used in our earlier studies (Zoladz et al., 2016; Jarmuszkiewicz et al., 2020), which analyzed the effects of the same endurance training procedure on other aspects of muscle and lung energetics in young rats. The western blots and tissue functional measurements (H_2_O_2_ production and enzyme activities) presented in this article were, for the purpose of this study, performed for the first time simultaneously in all tissues studied, as described below.

### 2.2 Tissue preparation

All homogenate preparation procedures were performed at 4 °C. The hearts, livers, brains (cortex), lungs, and hind limb muscles were harvested from control and exercised rats immediately after decapitation and placed in isolation medium A (50 mM Tris-hydrochloride [HCl; pH 7.2], 100 mM sucrose, and 0.5 mM ethylenediaminetetraacetic acid [EDTA]) and washed several times. After the organs were cleansed of larger blood vessels and surrounding tissue, they were shredded, and the remaining blood was removed by decantation. The tissues were homogenized in isolation medium B (50 mM Tris-HCl [pH 7.2], 100 mM sucrose, 1 mM monopotassium phosphate [KH_2_PO_4_], 100 mM potassium chloride [KCl], 0.5 mM EDTA, and 0.1 mM ethylene glycol-bis[β-aminoethyl ether]-N,N,N′,N′-tetraacetic acid using a polytron homogenizer (T18 basic; IKA-Werke GmbH & Co. KG, Staufen, Germany) for skeletal muscles (thrice for 2 s at 80% power) and a Teflon or glass pestle for the other organs. The homogenates were centrifuged at 900 *g* for 10 min. Protein concentration in the supernatants was measured using the Bradford method with bovine serum albumin as a standard.

### 2.3 Measurements of CS, COX, and lactate dehydrogenase activities

CS, COX, and lactate dehydrogenase (LDH) activities were all measured at 34 °C with constant stirring. CS activity was assessed by spectrophotometrically measuring the formation of 5,5′-dithiobis(2-nitrobenzoic)-coenzyme A (DTNB-CoA) from DTNB at 412 nm using a UV spectrophotometer (1620; Shimadzu Corp., Kyoto, Japan) in a reaction mixture (1 mL) containing 100 μg of protein homogenate, 100 mM Tris-HCl (pH 8.0), 0.1% Triton X-100, 100 μM oxaloacetate, 100 μM acetyl-CoA, and 100 μM DTNB.

LDH activity was measured by spectrophotometrically monitoring NADH oxidation (150 µM) at 340 nm in a reaction mixture containing 50–70 µg of protein homogenate, 20 mM pyruvate, and 50 mM Tris-HCl (pH 7.3).

The maximum COX activity was determined polarographically using a Clark-type oxygen electrode (Hansatech, King’s Lynn, United Kingdom)) in 0.7 mL of a standard incubation medium (225 mM mannitol, 75 mM sucrose, 10 mM KCl, 5 mM KH_2_PO_4_, 0.5 mM EDTA, 0.05% BSA, and 10 mM Tris-HCl [pH 7.2]) containing 5 mM ascorbate, 0.05% cytochrome *c*, and 40–70 µg of protein homogenate. Up to 1.5 mM N,N,N′,N′-tetramethyl-*p*-phenylenediamine (TMPD) was added sequentially to determine the maximum O_2_ uptake by COX.

### 2.4 Measurement of H_2_O_2_ production

The rate of H_2_O_2_ production was determined in 0.5 mL of standard incubation medium containing 50–100 µg of protein homogenate, 5 µM Amplex Red, 0.14 U/mL horseradish peroxidase (HRP), and 5 U/mL superoxide dismutase (SOD). Measurements were performed with 5 mM succinate, 5 mM malate, and 5 mM glutamate as respiratory substrates, in the presence of 0.5 mM ADP (phosphorylating conditions) and after its depletion (non-phosphorylating conditions). Fluorescence kinetics were monitored for 40 min at 34 °C, an excitation wavelength of 545 nm, and an emission wavelength of 590 nm using a microplate reader (Infinite M200 PRO; Tecan Group Ltd., Männedorf, Switzerland) and 24-well plates. H_2_O_2_ levels were quantified using a standard curve created with known amounts of H_2_O_2_.

### 2.5 Measurement of MDA levels

Lipid peroxidation was assessed by measuring MDA levels using the Amplite® Colorimetric Malondialdehyde (MDA) Quantitation Kit (AAT Bioquest, Pleasanton, CA, United States) according to the manufacturer’s protocol. Briefly, rat homogenates (150 µg of total protein adjusted to 50 µL) were incubated with 10 µL of MDA Blue™ in a clear-bottom 96-well microplate for 30 min. Then, 40 µL of the reaction solution was added. After a 30-min incubation, absorbance was measured at 695 nm using a microplate reader (Spark, Tecan Group Ltd., Männedorf, Switzerland). MDA levels were calculated using a standard curve created with known amounts of MDA.

### 2.6 Immunodetection of protein levels

Total proteins from rat organ homogenates were separated on 6%–12% sodium dodecyl sulfate-polyacrylamide gel electrophoresis gels, with the PageRuler Prestained™ Protein Ladder or Spectra™ Multicolor Broad Range Protein Ladder (Thermo Fisher Scientific) used as a marker of molecular weights. The following primary antibodies were used for immunodetection of target proteins: CS (46 kDa, ab96600), glutathione reductase (GR, 55 kDa) (ab128933), glyceraldehyde-3-phosphate dehydrogenase (GAPDH, 38 kDa, ab9485), transcription factor A, mitochondrial (TFAM, 28 kDa, ab131607), NRF2 (68 kDa, ab137550), SOD1 (18 kDa, ab13498), voltage-dependent anion channel 1 (VDAC1, 35 kDa, ab14734) (Abcam, Cambridge, United Kingdom), LDH (35 kDa, PA5-27406, Thermo Fisher Scientific, Waltham, MA, United States), hexokinase 1 (HK1, 120 kDa, sc80978, Santa Cruz Biotechnology, Dallas, TX, United States), and COX subunit 2 (COXII, 24 kDa, orb411834, Biorbyt, Cambridge, United Kingdom). GAPDH or actin (42 kDa, CP01; Merck, Darmstadt, Germany) was used for data normalization. Original, uncropped images with corresponding protein loading controls are shown in Supplementary Figures S1–S3. The protein bands were densitometrically analyzed using ImageJ software (US National Institutes of Health, Bethesda, MD, United States).

### 2.7 Statistical analysis

The data were statistically analyzed using Statistica (version 14.0; TIBCO Software, Santa Clara, CA, United States). The variables are presented as the mean ± standard deviation (SD) across 4–8 independent homogenate preparations. Normally distributed variables were compared between groups using an unpaired *t-*test or analysis of variance (ANOVA) followed by *post hoc* Tukey’s tests. Non-normally distributed variables were compared between groups using Kruskal–Wallis ANOVA (KW ANOVA) followed by Dunn’s *post hoc* comparisons. A *p* < 0.05 was considered statistically significant.

## 3 Results

### 3.1 Endurance training increases mitochondrial biogenesis in the lungs, brain, liver, skeletal muscle, and heart

**FIGURE 1:**
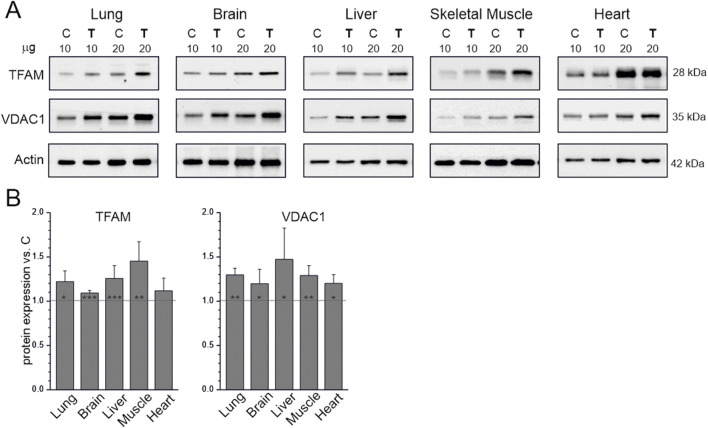
Mitochondrial biogenesis markers in tissues from control (C) and trained (T) rats. Representative western blots (cropped) **(A)** The μg values given refer to the amount of protein loaded into each lane. Mean ± SD of protein levels (*n* = 5) normalized to actin **(B)** Abbreviations: TFAM, transcription factor A, mitochondrial; VDAC1, voltage-dependent anion channel 1. Significance: *, *p* < 0.05; **, *p* < 0.01; ***, *p* < 0.001 vs. control (KW ANOVA).

We examined the effects of 8 weeks of endurance training on the levels of markers of mitochondrial biogenesis: TFAM, a key regulator of mitochondrial DNA transcription and replication (Kozhukhar and Alexeyev, 2023), and VDAC1, an outer mitochondrial membrane protein that regulates the exchange of metabolites between mitochondria and the cytoplasm (Camara et al., 2017). Endurance training increased TFAM and VDAC1 levels in all studied tissues (Figure 1); only the increase in TFAM in the heart was not statistically significant (Figure 1B).

### 3.2 Endurance training increases the oxidative capacity of mitochondria in the lungs, brain, liver, and skeletal muscles but not in the heart

**FIGURE 2:**
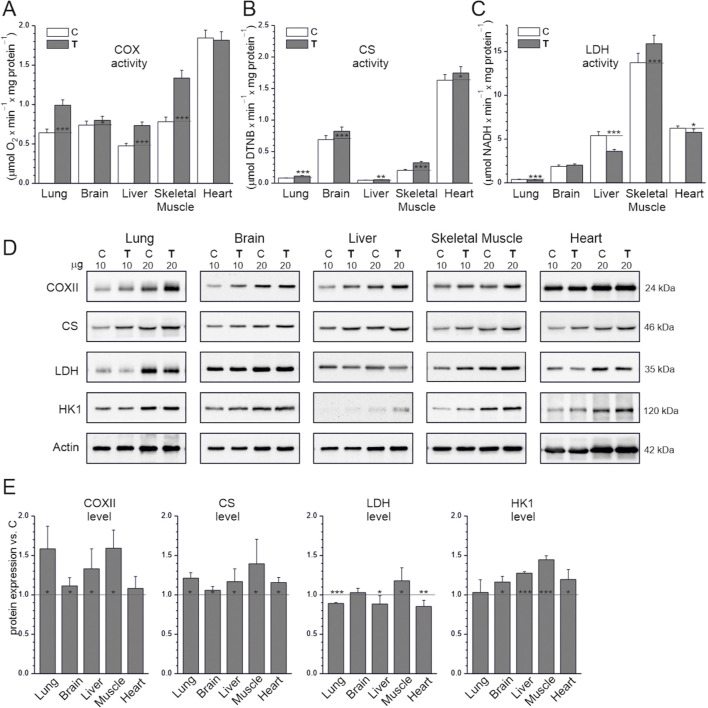
Maximal activities of key enzymes of aerobic respiration **(A,B)** and LDH **(C)**, representative western blots (cropped blots) **(D)**, and analysis of protein expression **(E)** in tissues from control (C) and trained (T) rats. The μg values given refer to the amount of protein loaded into each lane **(D)**. Abbreviations: DTNB, 5,5′-dithiobis(2-nitrobenzoic acid); CS, citrate synthase; COX, cytochrome *c* oxidase; COXII, COX subunit II; LDH, lactate dehydrogenase; HKI, hexokinase I. Protein expression levels were normalized to actin. **(A–C)** Mean ± SD, *n* = 8; statistics: one-way ANOVA. **(E)** Mean ± SD, *n* = 5; statistics: KW ANOVA. Significance: *, *p* < 0.05; **, *p* < 0.01; ***, *p* < 0.001 vs. control.

The levels of COX and CS, two other mitochondrial biogenesis markers, were also higher in the studied tissues in the endurance training group than in the sedentary control group, except in the heart, where COX levels were not significantly higher (Figure 2). Therefore, we examined the oxidative capacity of mitochondria by measuring the maximal activities of CS, an enzyme that regulates the rate of the Krebs cycle, and COX, which represents complex IV of the respiratory chain, in tissue homogenates obtained from both the sedentary control and endurance training groups (Figures 2A,B). Overall, maximal COX and CS activity in the studied tissues was higher in the endurance training group than in the sedentary control group, except for the heart, where only CS activity was higher. In the sedentary control group, COX and CS activity were at least 2.3-fold higher in the heart than in the other tissues. Notably, COX activity did not differ in the heart between the sedentary control and endurance training groups. Regarding COX activity, the difference between the sedentary control and endurance training groups was largest for skeletal muscle (∼70%), followed by the liver and lungs (∼50%), and smallest for the brain (∼8%; Figure 2A). Regarding CS activity, the difference was largest for skeletal muscle (∼60%), followed by the lung (∼43%), with smaller increases observed in the heart, liver, and brain (∼9%–19%), not differing significantly between these three tissues (Figure 2B).

The levels of HK1, the rate-limiting enzyme of glycolysis, were higher in the endurance training group than the sedentary control group in all tissues except the lung (Figures 2D,E). The difference in HK1 levels was greatest in the skeletal muscle (∼50%). We also investigated changes in the activity and expression of LDH, an enzyme responsible for converting pyruvate (the final product of glycolysis) into lactate, which can be reconverted into pyruvate and used as an oxidative fuel. In both the sedentary control and endurance training groups, LDH activity was highest in the skeletal muscles and lowest in the lungs (35-fold lower than in the muscles; Figure 2C). LDH activity and expression were higher in the endurance training group than in the sedentary control group in skeletal muscles but lower in the lungs, liver, and heart, and did not differ in the brain (Figures 2C–E). LDH activity was higher in the endurance training group than in the sedentary control group in the skeletal muscles (∼16%), lower in the heart and lungs (∼9%–16%), but much higher in the liver (∼33%).

These results indicate that endurance training increases the oxidative capacity of mitochondria in the lungs, brain, liver, and skeletal muscles but not in the heart. The upregulation in key respiratory enzymes is greater in skeletal muscle than in other tissues.

### 3.3 Endurance training increases ROS production in the heart and liver rather than in the skeletal muscles, lungs and brain, without leading to increased lipid peroxidation in any tissue. Even reduced lipid peroxidation in the lungs is observed after training

**FIGURE 3:**
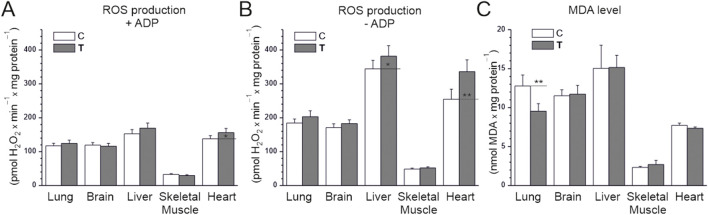
H_2_O_2_ production **(A,B)** and MDA levels **(C)** in tissues from control (C) and trained (T) rats. **(A,B)** measurements were performed in the absence or presence of ADP with malate, glutamate, and succinate as mitochondrial respiratory substrates. Mean ± SD, *n* = 6 **(A,B)**, *n* = 5 **(C)**; statistics: one-way ANOVA. Significance: *p* < 0.05 (*), *p* < 0.01 (**), comparison vs. control values for a given tissue.

We assessed the rate of H_2_O_2_ production in tissue homogenates under conditions of activation of the mitochondrial respiratory chain using a mixture of respiratory substrates (malate, glutamate, and succinate), providing electrons to complexes I and II. Measurements were performed with or without ADP, corresponding to the activation or deactivation of mitochondrial OXPHOS (Figures 3A,B). Comparing the examined tissues of untrained rats, the level of H_2_O_2_ production under OXPHOS activation conditions was highest in the liver and heart, slightly lower in the brain and lungs, and lowest in the skeletal muscles (at least 4 times lower) (Figure 3A). For all tissues, in the absence of ADP, H_2_O_2_ production was significantly higher compared to OXPHOS activation conditions (Figures 3A,B). Comparing the examined tissues of untrained rats, the level of H_2_O_2_ production in the absence of ADP was highest in the liver and heart and lowest in the skeletal muscles (Figure 3B). Namely, H_2_O_2_ production was ∼3.5 times higher in the brain and lungs, ∼5 times higher in the heart, and ∼7 times higher in the liver compared to skeletal muscle. In trained animals, a statistically significant increase in H_2_O_2_ production was observed in heart homogenates regardless of OXPHOS activation and in the liver under ADP-free conditions compared to untrained animals (Figures 3A,B).

We also measured the level of MDA, a marker of lipid peroxidation that indirectly indicates oxidative stress and antioxidant status. In the sedentary control group, MDA levels were lowest in skeletal muscles, ∼3.5-fold higher in the heart, and the highest in the brain, heart, and liver (∼5–7-fold higher than in skeletal muscle; Figure 3C). MDA levels did not differ significantly between the endurance training and sedentary control groups in all studied tissues except the lungs, where they decreased. Therefore, MDA levels did not change in tissues where ROS production was high without training (liver and heart), despite the increase in ROS caused by endurance training.

These results indicate that endurance training does not induce oxidative stress in the studied tissues, resulting in increased lipid peroxidation, but does increase ROS production in the heart and liver. Interestingly, endurance training reduces lipid peroxidation in the lungs, likely reducing oxidative stress.

### 3.4 Endurance training increases NRF2 and glutathione reductase levels in all studied tissues, with differential effects on SOD1

**FIGURE 4:**
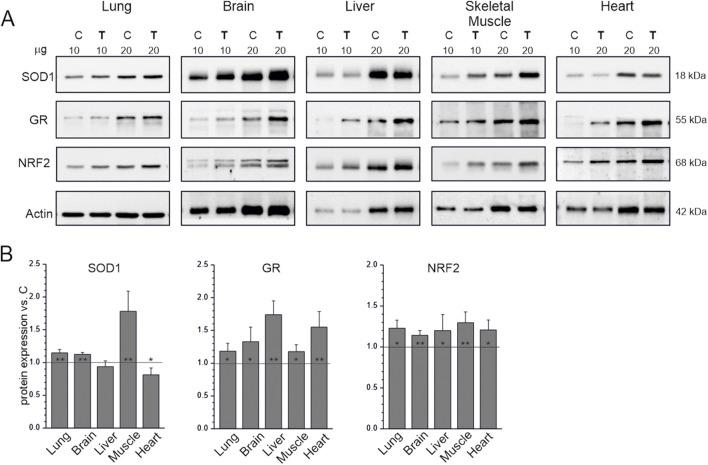
Antioxidant response markers in tissues from control (C) and trained (T) rats. Representative western blots (cropped blots) **(A)**. The μg values given refer to the amount of protein loaded into each lane. Mean ± SD of protein levels (*n* = 5) normalized to actin **(B)**. Abbreviations: SOD1, superoxide dismutase 1; GR, glutathione reductase; NRF2, nuclear factor erythroid 2-related factor 2. Significance: *, *p* < 0.05; **, *p* < 0.01; ***, *p* < 0.001 vs. control (KW ANOVA).

The levels of NRF2, which regulates the expression of genes involved in mitochondrial biogenesis, OXPHOS, and cellular antioxidant defense (Gureev et al., 2019), were higher in the endurance training group than in the sedentary control group in all studied tissues (Figure 4). Similarly, levels of the antioxidant enzyme glutathione reductase increased in all tissues of trained animals. Interestingly, in tissues where ROS production was higher in the endurance training group than in the sedentary control group (Figures 3A,B), SOD1 levels did not change (liver) or even decreased (heart), indicating no increased need for this antioxidant enzyme (Figure 4). In tissues where ROS production was lower than in the liver and heart in the sedentary control group, SOD1 levels were higher in the endurance training group, especially in the skeletal muscle (∼80%), where ROS production was the lowest among the studied tissues.

These results indicate that endurance training increased the activity of antioxidant defense in the studied tissues, as evidenced by NRF2 and glutathione reductase upregulation, to a level sufficient to avoid oxidative damage (lipid peroxidation).

## 4 Discussion

Our study showed that 8 weeks of endurance training elicited numerous adaptive responses in vital organs, including the brain, heart, lungs, liver, and skeletal muscle. This finding is consistent with recent reports indicating that the beneficial effects of physical activity/endurance training are not limited to skeletal muscle but extend to almost all body organs (Chow et al., 2022).

The applied endurance training increased the expression of factors that promote mitochondrial biogenesis, including TFAM and NRF2 (Figures 1, 4). Interestingly, endurance training did not increase TFAM levels only in the heart. Similarly, among the organs studied, only the heart did not show any increase in COX levels and activity after training (Figures 2A,E). The increase in VDAC1 expression in the heart without a corresponding increase in TFAM levels (Figure 1) may reflect adaptations related to increased metabolic activity and mitochondrial membrane transport induced by endurance training rather than a direct stimulation of TFAM-mediated mitochondrial biogenesis. These results indicate that the applied endurance training affected mitochondrial biogenesis differently in different organs. Specifically, the results indicate that mitochondrial biogenesis in the heart was more resistant to endurance training than in the other studied organs (skeletal muscle, lung, liver, and brain). Our results are consistent with recent studies by Khetarpal et al. (2025), who indicated that the mechanism of cardiac muscle adaptation to physical training appears to be different from that of skeletal muscle adaptation (Khetarpal et al., 2025). The authors demonstrated that cardiac adaptation to endurance training requires inhibition of growth differentiation factor 15 (GDF15), the expression of which is regulated by peroxisome proliferator-activated receptor gamma coactivator 1-α (PGC-1α). They highlighted the fact that, according to their studies, the adaptation of cardiomyocytes to physical training depends to a greater extent on PGC-1α than the adaptation of skeletal muscle (Khetarpal et al., 2025). These observations clearly indicate that the mechanisms of mitochondrial biogenesis during endurance training in different body organs may differ from those occurring in skeletal muscle. This may at least partially explain the differences in the regulation of mitochondrial biogenesis in skeletal muscle and heart that we observed in our study.

Among the organs studied, only the heart did not show any increase in COX levels and activity after training (Figures 2A,E). Regarding markers of tissue oxidative capacity, it has been argued that COX activity is the most reliable indirect marker of OXPHOS activity in tissues (Larsen et al., 2012; Zoladz et al., 2014). In our study, the maximal COX activity was similar in the lung, brain, and liver in the sedentary control group. Notably, that COX activity was significantly higher in the skeletal muscle and especially in the heart than in the other studied organs in the sedentary control group (Figure 2A). The applied endurance training significantly increased oxidative capacity in the hind limb muscles, as evidenced by the increase in maximal COX activity (∼70%; Figure 2A). This result is consistent with the previously described effects of endurance training on COX activity in hind limb skeletal muscles in rats (Holloszy, 1967) and in leg muscles in humans (Morgan et al., 1971; Wibom et al., 1992; Zoladz et al., 2022). Interestingly, in our study, we also observed that the applied endurance training significantly increased oxidative capacity in most studied organs (lungs, liver and brain) except, surprisingly, the heart (Figure 2A). Notably, the endurance training-induced increase in COX activity was considerable and similar in the liver and lung (∼50%) but much smaller in the brain (∼8%; Figure 2A). Our results indicate that in young animals, organs in which endurance training has no or minimal effect on oxidative capacity (brain and heart) have sufficient reserves of oxidative capacity to withstand the load of endurance training without the need to increase its level, unlike the other studied organs (lungs, liver and skeletal muscle).

In our study, we observed systematically lower ROS production in all tissues (lung, brain, liver, skeletal muscle, and heart) under phosphorylating conditions (in the presence of ADP) than under non-phosphorylating conditions (in the absence of ADP; Figures 3A,B). These findings are consistent with previous studies that showed that H_2_O_2_ production in isolated skeletal muscle mitochondria was several times higher in the non-phosphorylating state (state 4) than in the phosphorylating state (state 3) (Jarmuszkiewicz et al., 2015; Zoladz et al., 2016). Another study also addressed this issue (Korshunov et al., 1997), postulating that the high proton force in state 4 is potentially dangerous for the cell due to the increased probability of superoxide formation. Assuming, as a rough approximation, that state 3 respiration is closer to the functioning of muscle mitochondria during muscular activity and state 4 respiration is closer to the functioning of mitochondria at rest (Kavazis et al., 2009; Goncalves et al., 2015), this observation suggests that tissues such as skeletal muscle and heart, which dramatically increase their metabolism during exercise (Zoladz et al., 2016), are exposed to lower ROS production by mitochondria during exercise than at rest. Based on our results (Figures 3A,B), it appears that mitochondrial ROS production is much lower under phosphorylating conditions than under non-phosphorylating conditions also in other tissues, including the lung, brain, and liver. They are consistent with the view that mitochondria are not the primary sources of H_2_O_2_ in working muscles (Powers et al., 2024b; 2024a). Therefore, NADPH oxidase 2 (NOX2) is considered the primary source of ROS during physical exercise (Henríquez-Olguin et al., 2019).

It is also worth noting that in our study, mitochondrial H_2_O_2_ production in the heart, measured under both phosphorylating and non-phosphorylating conditions, was several times higher than in skeletal muscle, regardless of training status (sedentary vs. endurance training group) (Figures 3A,B). Similarly, the MDA level in the heart was also several times higher than in skeletal muscle (Figure 3C). These findings are consistent with our previous report, which showed that glutathione disulfide content in the heart at basal state was significantly higher than in skeletal muscle (Majerczak et al., 2022). Together, these results suggest that the heart, even at rest, is exposed to greater oxidative stress than resting skeletal muscle. This may be explained by the fact that the metabolic rate of the heart at rest, when expressed per unit of tissue mass, is several times higher than that of resting skeletal muscle (Zoladz et al., 2015).

Endurance training has previously been shown to significantly increase H_2_O_2_ production in isolated rat skeletal muscle mitochondria under non-phosphorylating conditions and to decrease their H_2_O_2_ production under phosphorylating conditions (Zoladz et al., 2016). In our study, we found that the applied endurance training had almost no effect on ROS production under phosphorylating conditions in the studied tissues, except in the heart, where H_2_O_2_ production was higher after training (Figure 3A). Interestingly, H_2_O_2_ production under non-phosphorylating conditions in the heart and liver was higher in the endurance-trained group than in the sedentary control group (Figure 3B). The observed endurance training-induced increases in H_2_O_2_ production in the heart (both under phosphorylating and non-phosphorylating conditions) and liver (only under non-phosphorylating conditions) could be traditionally interpreted as a potentially harmful effect of endurance training on these tissues (Davies et al., 1982). However, given the increasing evidence, this response (training-induced increase in mitochondrial H_2_O_2_ emission) should instead be interpreted as training-induced activation of redox signaling pathways, which seem to play a key role in tissue adaptation to endurance training (Powers et al., 2024b; Powers et al., 2024a).

It is well established that SODs are the first line of defense against free oxygen radicals, and most organisms living in the presence of oxygen express at least one SOD isoform (Wang et al., 2018). Since SODs are the only enzymes that interact specifically with superoxide anion and thus control ROS and reactive nitrogen species levels, they also act as key regulators of cell signaling. Mammals possess three SOD isoforms (SOD1, SOD2, and SOD3), of which SOD1, acting in the largest compartment of the cell, including the cytoplasm and the intermembrane space of mitochondria, appears to play a key role in the antioxidant system. In our study, we found that the applied endurance training had different effects on SOD1 expression in the studied tissues. Specifically, we observed increased SOD1 levels in the lung, brain, and skeletal muscle, where the increase was greatest (Figure 4). However, no changes in the liver, and even a reduction in SOD1 levels in the heart, were observed after endurance training. These results indicate that endurance training differentially affects the SOD1-related antioxidant defense mechanism in different organs. Notably, both the heart and liver displayed higher ROS production than the other organs studied, both before and after training. This observation suggests that these organs are under greater oxidative load and may therefore rely on organ-specific regulatory strategies to maintain redox balance. In the heart, the reduction in SOD1 levels may be offset by increased activity of other antioxidant enzymes, such as glutathione reductase (Figure 4), while the liver may sustain its defenses without altering SOD1. Therefore, the observed decrease in SOD1 levels in the heart does not necessarily indicate reduced protection but may reflect compensatory adjustments tailored to ROS management in this organ. Interestingly, the applied endurance training did not increase lipid peroxidation in the studied tissues, as assessed by MDA levels in their homogenates (Figure 3C). In the heart and liver, the increase in H_2_O_2_ production was not accompanied by elevated MDA levels, which may indicate the involvement of compensatory antioxidant pathways such as catalase, peroxiredoxins, or the glutathione antioxidant system (as shown by glutathione reductase, Figure 4). Another possibility is that ROS production occurred in specific subcellular compartments, where localized increases in H_2_O_2_ did not translate into detectable systemic oxidative damage. However, our results indicate that endurance training sufficiently increased antioxidant defense activity in all studied tissues (lung, brain, liver, skeletal muscle, and heart), as assessed by the observed increases in NRF2 levels in all studied tissues (Figure 4). The intensification of systemic or organ antioxidant activity after training is a well-document physiological response (Powers and Jackson, 2008; Powers et al., 2024b; Powers et al., 2024a). Indeed, numerous studies have shown an upregulation of antioxidant activity in various organs, including skeletal muscle, brain, liver, and heart (for reviews see (Goto and Radák, 2009; Powers et al., 2024b; Powers et al., 2024a). This physiological adaptive response is considered to be beneficial to the body because it attenuates the potentially harmful effects of exercise-induced ROS production. On the other hand, supplementation with antioxidants (e.g., vitamins C and E) has been shown to attenuate the beneficial effects of training, including preventing exercise-induced antioxidant expression and attenuating/preventing enhanced mitochondrial biogenesis in the time course of training (for reviews see (Hamilton et al., 2003; Gomez-Cabrera et al., 2008; Goto and Radák, 2009; Ristow et al., 2009; Powers et al., 2024b; Powers et al., 2024a). These studies have led to the conclusion that mild oxidative stress induced by exercise is actually beneficial to the body because it stimulates a number of adaptive responses, including upregulation of the antioxidant system, resulting in reduced muscle damage and increased mitochondrial biogenesis. This concept (the hormetic effect of ROS) revealed the other side of exercise-induced ROS production (Goto and Radák, 2009; Powers et al., 2024b; Powers et al.,2024a). However, the effect of exercise on redox balance in various organs is still poorly understood.

There are convincing reports that NRF2 plays a key role as a transcription factor that regulates cellular defense against toxic and oxidative attacks by modulating the expression of genes involved in oxidative stress response and drug detoxification (He et al., 2020; Gao et al., 2021; Esteras and Abramov, 2022; Powers et al., 2024a; Zhou et al., 2024). Therefore, through its involvement in metabolic reprogramming, unfolded protein response, proteostasis, autophagy, mitochondrial biogenesis, inflammation, and immunity (He et al., 2020; Gao et al., 2021; Esteras and Abramov, 2022; Powers et al., 2024a), NRF2 seems to play a key role in adaptation to physical exercise. Our study is the first to our knowledge to demonstrate that endurance training increases NRF2 expression in the lung, brain, liver, skeletal muscle, and heart (Figure 4). It is intriguing that, as demonstrated in the present study, physical training effectively increased NRF2 expression simultaneously in multiple organs, including the lungs, brain, liver, skeletal muscle, and heart. It is not clear whether and how the signal informing about oxidative stress occurring in a specific organ of the body, e.g., in skeletal muscles during exercise, is transmitted to another organ in order to build an adaptive stress response (Sies, 2024). We cannot exclude the possibility that the multi-organ upregulation of NRF2 expression in response to physical training, as observed in our study, is the result of intercellular or inter-organ communication, a mechanism recently postulated by Sies et al. (Sies, 2024; Sies et al., 2024). We postulate that the endurance training-induced increase in NRF2 levels in various organs may play a key role in the observed beneficial effects of physical activity on the functioning of various organs in the human body (Pedersen and Saltin, 2015; Warburton and Bredin, 2017; Warburton and Bredin, 2019).

In this study, we examined for the first time the effects of endurance training on mitochondrial H_2_O_2_ production under phosphorylating and non-phosphorylating conditions, as well as on NRF2 levels in multiple vital organs (lungs, brain, liver, heart, and hind limb skeletal muscle) within the same animals. A major strength of this work lies in its comparative approach, which revealed tissue-specific differences in ROS production and antioxidant responses to endurance training. By integrating measurements of ROS generation, oxidative damage, and antioxidant enzyme expression, the study provides an organ-level overview of redox regulation under physiological stress. Nevertheless, several limitations should be acknowledged. The study was conducted exclusively on young adult male rats, limiting extrapolation to females or older animals. Further studies are needed to assess sex-dependent differences and to explore the impact of aging on mitochondrial ROS production and NRF2-modulated antioxidant defenses in different organs. Although five key tissues were analyzed, other organs with high oxidative susceptibility, such as the kidneys and intestine, were not included. NRF2 activation was assessed indirectly, based on protein levels of selected downstream enzymes (SOD1, glutathione reductase), without assessment of NRF2 nuclear translocation or a broader set of NRF2 signaling targets, which are central to the antioxidant response (e.g., heme oxygenase 1 (HO1), NAD(P)H quinone dehydrogenase 1 (NQO1), catalase, or peroxiredoxins). Moreover, compartment-specific ROS production and direct measurements of mitochondrial oxidative capacity or OXPHOS protein expression were not included. Future studies should expand the range of tissues, include both sexes and older animals, and apply more detailed analyses of NRF2 signaling, compartmental ROS dynamics, mitochondrial function, and total antioxidant capacity. Such work would provide deeper mechanistic insight into organ-specific redox responses to endurance training.

## 5 Conclusion

Our study showed that endurance training increased oxidative potential in skeletal muscle and in all studied organs (lung, liver, and brain) except the heart. These results indicate that, compared to other studied tissues, the heart of a young animal, which is already characterized by the highest oxidative activity before training, has sufficient ATP production capacity to maintain endurance without upregulating the oxidative ATP supply system. However, endurance training significantly increased mitochondrial H_2_O_2_ production in the heart (both under phosphorylating and non-phosphorylating conditions) but not in the other studied tissues, except for the liver, where it was increased under non-phosphorylating conditions after endurance training. This observation indicates that physical exercise induced greater activation of H_2_O_2_-related cell signaling in the heart than in the other studied tissues. The most interesting and, to our knowledge, original finding of our study was the observed endurance training-induced increase in NRF2 levels in all studied tissues (brain, heart, lung, liver, and hind limb skeletal muscle). Given the cytoprotective properties of NRF2 discussed above, we believe that this multi-organ adaptive response plays a key role in the widely described beneficial effects of physical activity on various body organs and organismal health.

## Data Availability

The original contributions presented in the study are included in the article/Supplementary Material, further inquiries can be directed to the corresponding authors.
