# Supplementary material for: Impact of endurance training on mitochondrial H2O2 production and NRF2 levels in different rat organs
Source: Front Mol Biosci. 2025 Oct 16;12:1653162. doi: 10.3389/fmolb.2025.1653162 (PMC12571570; doi:10.3389/fmolb.2025.1653162)
Supplement: Supplementary file 1 [file DataSheet1.pdf]

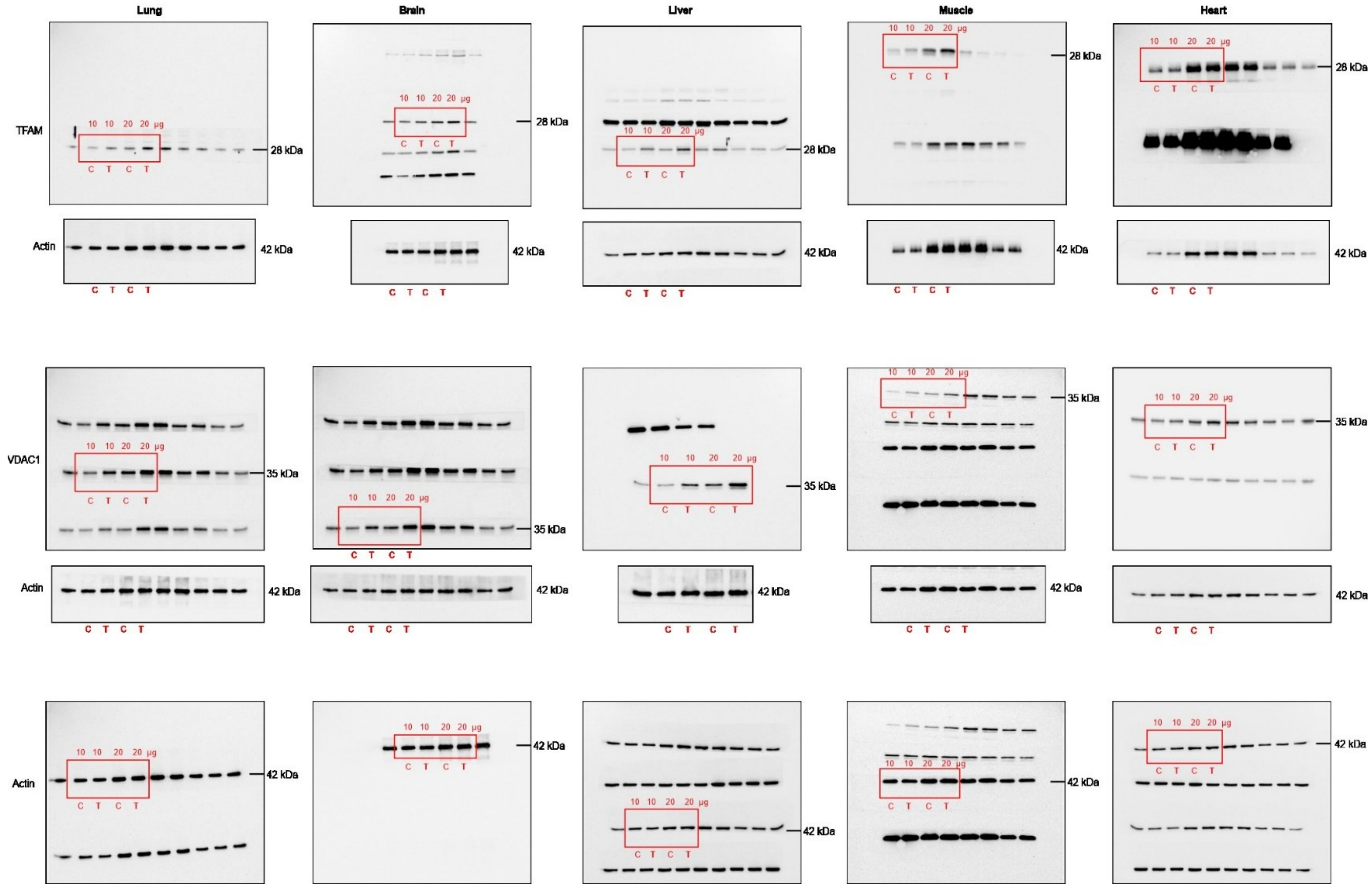

Fig. S1. Uncropped images used for preparation of Fig. 1 with corresponding protein loading controls (actin)

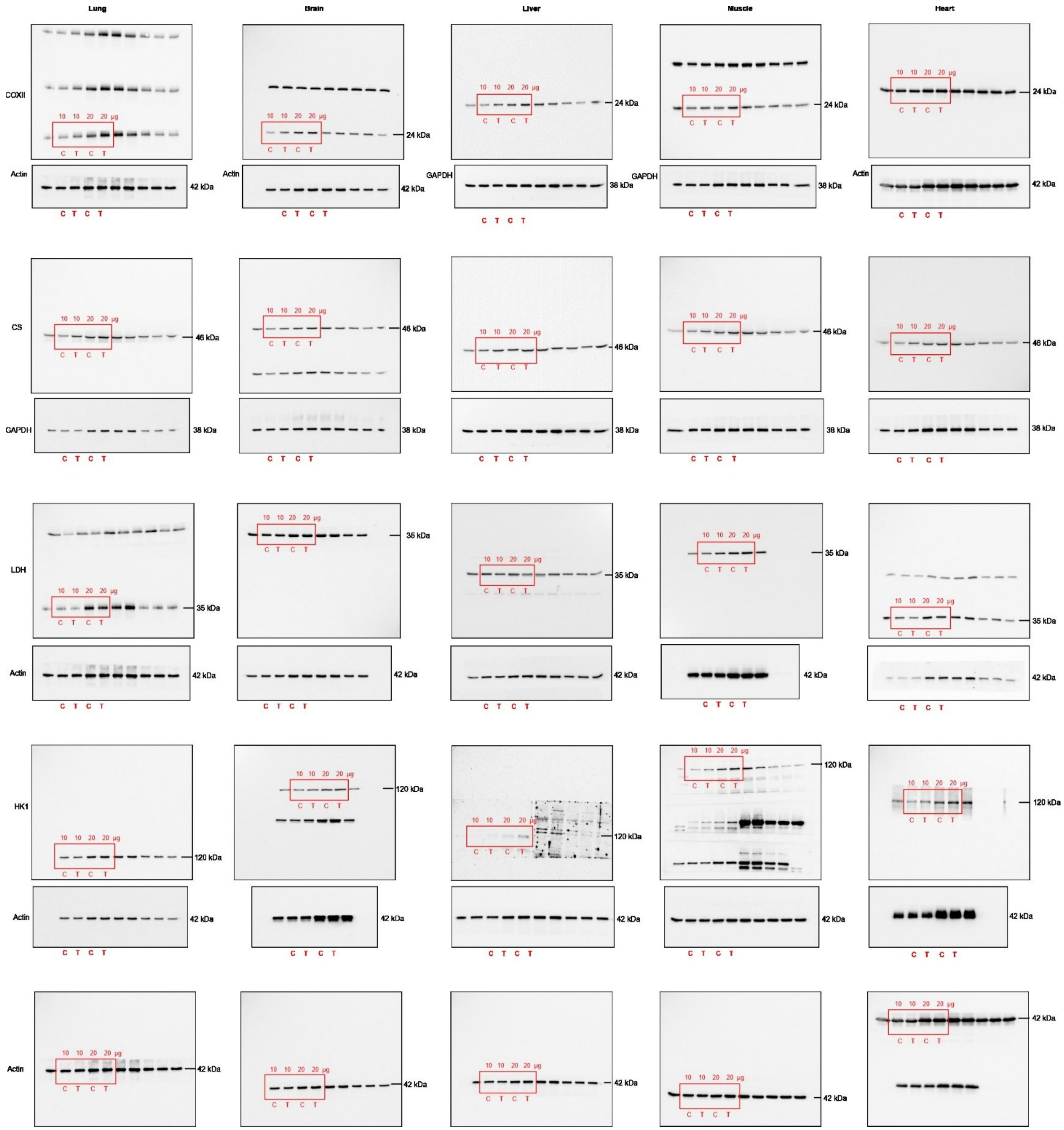

Fig. S2. Original images used to prepare Fig. 2 with relevant protein loading controls (actin or GAPDH)

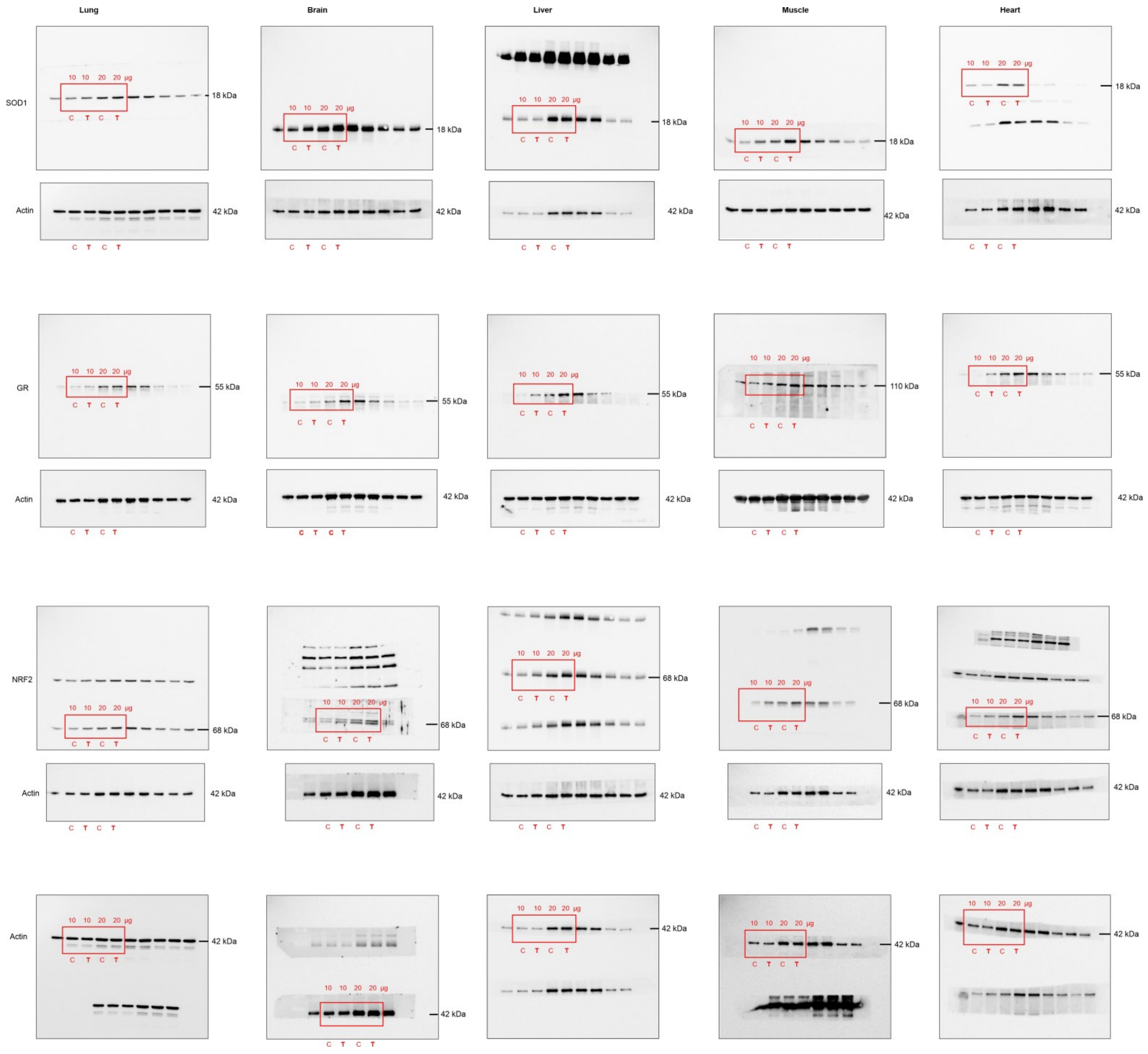

Fig. S3. Original images used for preparation of Fig. 4 and associated protein loading controls (actin)
